# Quality Control Approach for the Detection of Internal Lower Density Areas in Composite Disks in Industrial Conditions Based on a Combination of NDT Techniques

**DOI:** 10.3390/s21217174

**Published:** 2021-10-28

**Authors:** Andrzej Katunin, Krzysztof Dragan, Tomasz Nowak, Marek Chalimoniuk

**Affiliations:** 1 Department of Fundamentals of Machinery Design, Silesian University of Technology, Konarskiego 18A, 44-100 Gliwice, Poland; 2 Division of Airworthiness, Air Force Institute of Technology, Ks. Bolesława 6, 01-494 Warsaw, Poland; krzysztof.dragan@itwl.pl; 3 Hitachi ABB Power Grids, Pawia 7, 31-154 Kraków, Poland; tomasz.nowak@hitachi-powergrids.com; 4 Aircraft Engines Division, Air Force Institute of Technology, Ks. Bolesława 6, 01-494 Warsaw, Poland; marek.chalimoniuk@itwl.pl

**Keywords:** non-destructive testing, voids, manufacturing defects, composites manufacturing, ultrasonic testing, infrared thermography, X-ray computed tomography, defects detectability

## Abstract

Voids in polymer matrix composites are one of the most common manufacturing defects, which may influence the mechanical properties and structural behavior of the final parts made of composites by various manufacturing methods. Therefore, numerous non-destructive testing (NDT) techniques were developed and applied for quality control and in-service testing of such structures. In this paper, the authors analyzed various alternatives to the reference technique, X-ray computed tomography (XCT) NDT, which is used for industrial testing of composite disks having defects in the form of the lower density areas. Different candidates, namely: vibration-based testing, infrared thermography, vibro-thermography, as well as ultrasonic testing were analyzed in terms of their sensitivity and technical feasibility. The quality of the results, the complexity of the testing procedure, time and labor consumption, and the cost of the equipment were analyzed and compared with the reference technique. Based on the conducted research the authors finally proposed a hybrid approach to quality control, using a combination of two NDT techniques–infrared thermography (for initial scanning and detection of near-surface defects) and ultrasonic testing (for a more detailed analysis of products that pass the first testing procedure). It allowed for replacing the costly XCT diagnostics with a much cheaper, but almost equally effective, alternative.

## 1. Introduction

Voids occurring in polymeric and polymer matrix composite (PMC) structures during their manufacturing belong to the class of manufacturing defects that appear in such structures most often. Their appearance is typical for all manufacturing methods being used for polymeric and PMC structures. The reasons for such phenomenon are usually related to inappropriate processing parameters; for example, incorrectly set temperature and pressure cycles (causing boiling of a polymer), a flow speed of a liquid polymer and its viscosity during resin infusion (RI) or resin transfer molding (RTM) manufacturing techniques [1,2,3,4,5], the cumulation of entrapped gas bubbles, an unevenly distributed temperature field in the volume of an injected liquid polymer [6], etc. Moreover, connecting the mentioned reasons with the manufacturing process, the appearance of voids in polymeric and PMC structures can be also related to the specificity of a processed polymer, i.e., nucleation processes occurring during crystallization or solidification, the influence of residual volatiles, dissolving or absorption of moisture and other gases during the manufacturing process, etc. [7,8]. Depending on the type of processed polymer, the nature of the formation of voids is different: while in thermoplastics the formation of voids is a result of curing or, in general, processing itself, the formation of voids in thermosetting polymers is largely dependent on the defects present before a curing process [9].

Undoubtedly, the presence of voids in polymeric and PMC structures causes a significant influence on their numerous properties. One of the main deficiencies of a polymeric or PMC structure containing internal voids is the degradation of its mechanical properties, including flexural, compressive and shear strength, fatigue resistance, and fracture toughness (which is directly related with the fundamental Griffith’s concepts of fracture mechanics [10]), confirmed in numerous studies [2,8,11,12,13,14,15,16,17]. Consequently, the local inhomogeneity caused by the presence of voids also influences the modal parameters of the entire structure, which was confirmed by Yang et al. [18].

The influence of voids on the mentioned properties is governed mainly by their morphology, location, content, and distribution in a volume of a structure [8,19,20,21,22,23]. During operation, the presence of voids creates ideal conditions for nucleation of micro-scale damage, such as cracks and fibrils, which is a result of material inhomogeneity, creating the stress concentration in the surrounding voids. This, in turn, results in void coalescence as well as crack initiation and propagation [20,24,25,26], which leads to premature structural failure caused by static and/or fatigue loading of a structure. Moreover, in addition to the above-described purely mechanical nature of propagation of nucleated cracks from internal voids, another propagation mechanism is possible. In the case when a strong electric field interacts with entrapped gases or water in internal voids, partial discharges appear in their location, launching local chemical degradation mechanisms. This in turn makes the material locally conductive, and as a result of the prolonged action, causes the initiation and propagation of mechanical damage, primarily in the form of cracks [27,28,29].

According to the above-discussed negative influence of voids on the mechanical properties of structures and parts made of polymers and PMCs, the effective methods of their detection and localization at the production inspection (PI) and quality control (QC) stages, as well as inspection and monitoring during the operation of polymeric or PMC structures are necessary. Many such methods, which are mostly based on non-destructive testing (NDT) techniques, are successfully used in engineering practice. Initially, as it was stated by Pawlak et al. [30], the methods of void detection fall into three groups: microscopic testing, electromagnetic radiation scattering, and evaluation of density or volume changes. These methods, in general, have numerous limitations and provide only qualitative information and a high level of uncertainty of the presence of voids in a tested structure. Moreover, in the case of microscopic testing, preparing the cross-sections of a material is necessary, e.g., to evaluate the volume fractions of voids [8], which excludes this approach from the class of NDT techniques.

Among the currently applied NDT techniques that found wide application in numerous studies, one can mention, among others, ultrasonic testing (UT), radiographic testing (RT) and X-ray computed tomography (XCT), and infrared thermography (IRT), as the most popular ones in the detection and identification of internal defects and damage due to their sensitivity to these types of flaws [6,8,31]. The overviews on other NDT techniques can be found in the above-cited review papers. The principles of defects detection of these techniques are as follows: UT is based on the analysis of the propagation of high-frequency sound waves in a tested material, and further evaluation of a flaw presence and position based on the reflection of sound waves from this flaw. IRT uses a phenomenon of irradiation of a tested object in the infrared range, previously excited thermally, and based on local differences in temperature distribution due to disturbed heat conduction at flawed regions, and the identification of these regions is possible. RT and XCT belong to the same group of NDT techniques based on the passage of X-ray radiation through a tested object and registering a result from its backside. The identification of flaws is possible due to disturbed passage of radiation through the flawed regions, which is visible in resulting patterns. The main difference between RT and XCT is that RT results in a 2D image, while with XCT, obtaining results in the form of a 3D array is possible. However, the mentioned techniques are characterized by different sensitivity and detectability of voids. Undoubtedly, the most effective approach is based on XCT, which provides very high sensitivity (industrial XCT allows reaching the lowest resolution of micrometers to tens of micrometers) as well as the ability of three-dimensional imaging of voids. The effectiveness of this approach is confirmed both by Nsengiyumva et al. in their comparative study of the performance of NDT methods in the detection of various types of defects and damage [6] as well as in numerous other studies [23,26,32,33,34,35,36,37,38]. Nevertheless, the main drawback of XCT is a limitation regarding the dimensions of tested objects (which is additionally connected to its spatial resolution) as well as the relatively high cost of testing equipment and time-consuming testing procedures. The group of RT techniques, although revealing some sensitivity to voids [6,39], generate a radiation hazard and require a specific surrounding in which the testing can be safely performed.

From the above-mentioned NDT techniques, it is essential to pay attention to UT, which does not provide highly accurate information on void morphology and dimensions as XCT due to a generally lower resolution (the spatial resolution of UT varies from hundreds of micrometers to millimeters depending on the scanning system and scanning parameters), but constitutes one of the main NDT industrial tools for such inspections [8]. Moreover, the costs of UT inspections are significantly lower compared to XCT and there are no limitations regarding the dimensions of the tested object within this technique. The high sensitivity to voids is confirmed by Nsengiyumva et al. in [6] and in the review paper by Birt and Smith [40], where the authors analyzed NDT methods for the detection of porosity in PMCs. Successful detection and localization of voids in PMCs were confirmed in [41], where the authors evaluated the void content based on the obtained C-scans using the double through-transmission UT as well as in [42], where multiple voids were detected in hybrid glass-carbon fiber-reinforced PMCs using the angle beam UT inspection technique. The authors of [43] used sampling phased array UT detection of voids in carbon-fiber-reinforced PMCs with much success. The results of UT inspections are usually presented in the form of colormaps (in the case of B- and C-scans), representing information on a planar projection of the damage shape and dimensions as well as its depth represented by a color. Recent advances [44,45] show that UT results can be reconstructed in 3D to obtain enhanced information about the damage position.

The next promising technique among the widely used NDT techniques is the IRT, which is a technique that is relatively inexpensive, portable, and non-contact [8,33], and reveals a sensitivity to voids in polymeric and PMC structures comparable to UT [6]. The resolution of IRT is usually in the range of millimeters, however, there are micro-IRT systems available, where resolution is on the level of tens to hundreds of micrometers. The simple physics behind IRT, namely the determination of changes in thermal conductivity or diffusivity, makes it fast and easy to use. The application of IRT to the detection and localization of voids in polymeric and PMC structures was confirmed both in laboratory [46,47] and in-field conditions [48] using various IRT setups. Among the variety of IRT techniques, one can mention the newly developed vibrothermographic methods [49,50], including the self-heating vibrothermograhy (SHVT) described in [51,52], which, due to external excitation and thus local heating, are able to detect various types of internal damage in polymeric and PMC structures, including voids.

Among the other methods which found application in the detection of voids in polymers and PMCs, one can distinguish a class of terahertz testing (TT) techniques with a resolution on a submillimeter level. This includes terahertz time-domain spectroscopy and a frequency modulation continuous wave method, which seems to be a promising approach for such a class of problems with prior proven effectiveness [6,8,53,54,55,56,57,58]. However, the experimental setup is usually very complicated and costly, while the penetration capability is insufficient in the case of thick structures [6], as well as the technique is limited to electrically inactive polymers.

Besides the traditional and widely applied methods, one can find numerous studies on the application of unconventional NDT techniques for the detection and localization of voids in polymeric and PMC structures. The authors of [59] detected voids based on the vibration response of the tested polymeric and PMC structures. The idea of analyzing vibration responses is based on the observation of changes in frequency response functions, as well as changes in mode shapes discussed, e.g., in [60,61,62]. The observable changes are connected with the changes of a local structural stiffness caused by the presence of damage, in particular, internal voids. Several review papers also mention the application of shearography [8] and optical densitometry [63], however, their performance is not sufficiently confirmed.

The aim of the performed research studies was the development of a new hybrid approach to QC, as an alternative to the reference XCT technique, based on particular NDT methods for the detection of lower density areas in the composite structures of complex geometry. Selection of the most promising NDT techniques to be combined in a single methodology for PI and QC of the investigated composite disks was performed based on the reference measurements using XCT, and considering the industrial requirements, namely, the speed of inspection as well as the complexity of performing measurements and the costs of the testing apparatus.

Keeping in mind the above-presented criteria as well as the overview based on published research studies, it was possible to perform a preselection of NDT techniques applicable for the defined task. From the above-presented analysis, it is clear that the most sensitive and accurate technique is XCT, which is confirmed in numerous studies (see the overview on XCT presented in [6]). However, due to the very high cost of inspection as well as the impossibility of testing big objects (and a significant decrease in spatial resolution with the increase in the dimensions of the tested object), this technique was excluded from consideration as the reliable NDT technique for PI and QC. Nevertheless, due to the mentioned advantages, this technique was considered as the reference one to evaluate the sensitivity of the preselected NDT techniques. Similar to XCT, the group of RT techniques was also excluded from the set of considered reliable NDT techniques for PI and QC due to the hazardous influence on the operator and the necessity of the construction of an isolated area for performing NDT inspections. The TT technique seems to be a promising candidate for the defined task, as it was mentioned before, but was also excluded due to the very high cost of the testing apparatus and the complexity of its maintenance. From the above-considered techniques, both UT and IRT were selected due to good and comparable sensitivity to lower density areas in the tested disks, relatively low costs compared to the previously considered methods, as well as simplicity in performing of the tests, ease of interpretation of results, and maintenance of a testing facility. Moreover, these methods were selected due to the relatively good sensitivity to voids in thick polymeric and PMC structures, which was confirmed in the comparative study presented in [6]. Furthermore, of these basic methods, the vibration-based approach was considered as the initial technique tested within this study as well as the newly developed SHVT approach described above.

The main goal of this study is to analyze and compare the preselected NDT techniques, verify their sensitivity based on the results of the XCT reference scans, and develop a combined approach for PI and QC of composite elements based on preselected NDT techniques. The possibility of the adaptation of the developed approach to industrial application conditions was discussed. Most of the NDT studies available in the literature are focused on the detection and identification of specific defects or damage using specific NDT techniques, while the presented study is focused on providing general recommendations for the application of the preselected NDT techniques in industrial conditions, which fills a gap in the available literature in this area. The results of this study might be interesting especially for NDT industrial practitioners, as well as scientists working in this thematic area.

## 2. Materials and Methods

### 2.1. Tested Structures

**Figure 1:**
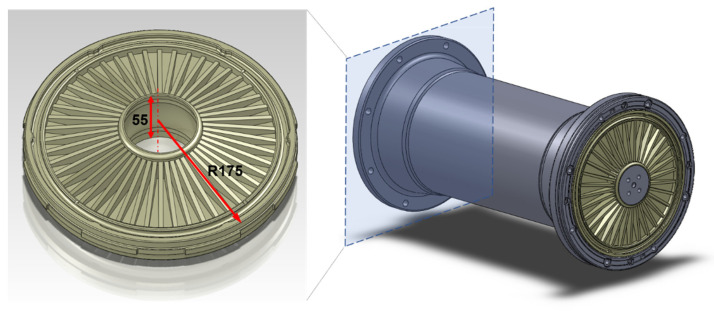
Schematic models of a barrier insulator (**left**) used in GIS ducts (**right**).

The structures subjected to testing procedures represented insulation elements of Gas Insulated Substations (GIS)—vital nodes of the power transmission network. The functional sections of GIS solutions are separated by insulators having the form of a disk. A small, cylindrical part in the center of the disk is made of metal and it conducts electric current, while the main insulating part, reinforced with ribs, is usually made of epoxy resin or ceramics (see the schematic view in Figure 1 with main dimensions).

**Figure 2:**
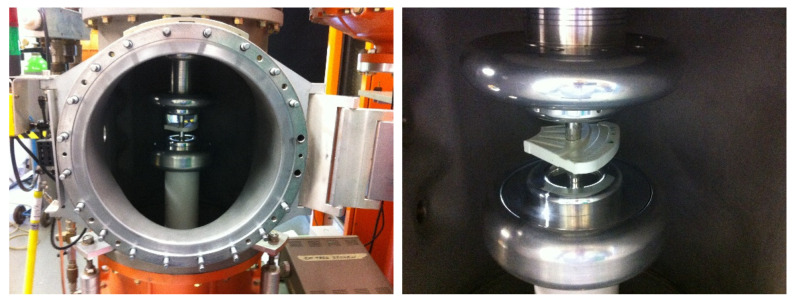
The barrier insulator disks during the electrical breakdown strength testing.

During normal operation, disk insulators are subjected to mechanical loading—they support the weight of the conductive bars and transfer electromagnetic forces to the enclosure. Additionally, during maintenance of particular GIS sections, insulators are bent due to gas pressure acting only on one side of the disk. From a dielectric viewpoint—the object is exposed to a strong electric field, which is manifested by partial discharges arising from defects in the material, such as voids or pores. The evaluation of the electrical response of the tested disks was a subject of previous tests, including the electrical breakdown strength. The tests showed that, depending on the applied voltage and the type of insulation gas, the critical size of defects causing dielectric problems ranges from 0.2 to 0.7 mm. The setups of these tests are presented in Figure 2.

Normally, barrier insulators are manufactured using casting technology. Yet, recent achievements in the field of composite materials (such as epoxy resin-coated silica filler composites) and advances in material processing technology allowed for significant progress in the production of barrier insulators, which found an application in manufacturing the tested objects in this study. However, in this case, the quality of the manufacturing plays an even more important role. It is driven by the fact that the thickness of walls is limited due to technological and processing restrictions. On the other hand, the product cannot simply be thickened or enlarged to compensate for any defects. Therefore, both the manufacturing process and final products must exhibit high quality. In this context, the need for effective NDT methods that ensures high-quality production is evident.

**Figure 3:**
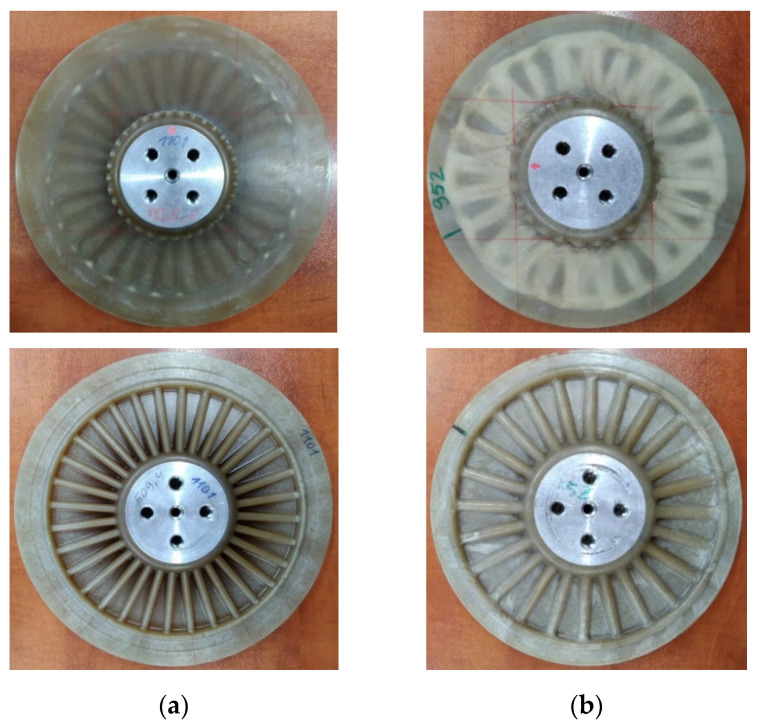
The top and bottom views of the tested disks: (**a**) The larger disk; (**b**) The smaller disk.

In this study, to test the applicability of various NDT techniques and their sensitivity to the investigated types of damage, two disks were taken into consideration: the first one, further called the larger disk with a diameter of 230 mm (see Figure 3a), and the second one, further called the smaller disk with the diameter of 210 mm (see Figure 3b). While the larger disk was manufactured within a typical manufacturing process, the smaller disk was manufactured with intentionally changed manufacturing parameters to simulate the investigated lower density areas, which are well visible in Figure 3b in the form of lighter regions.

### 2.2. Applied Non-Destructive Testing Techniques

Four NDT techniques, IRT, SHVT, and UT, which are the damage identification based on modal analysis, were preselected for testing of the considered composite disks with internal lower density areas. Additionally, XCT was the fifth technique used in this study as the reference for the evaluation of the performance of the preselected NDT techniques. Moreover, this technique provides a high-resolution capability for structural integrity evaluation due to the possibility of volumetric inspection of a material. A detailed description of the experimental setups for these techniques is presented in this section.

#### 2.2.1. Damage Identification Based on Modal Analysis

The vibration-based testing was considered in this study due to a potential possibility of detection and localization of the above-mentioned defects (see Section 2.1) by applying post-processing procedures to the determined mode shapes. This approach was proven in numerous previous studies (see e.g., [60,61,62]).

**Figure 4:**
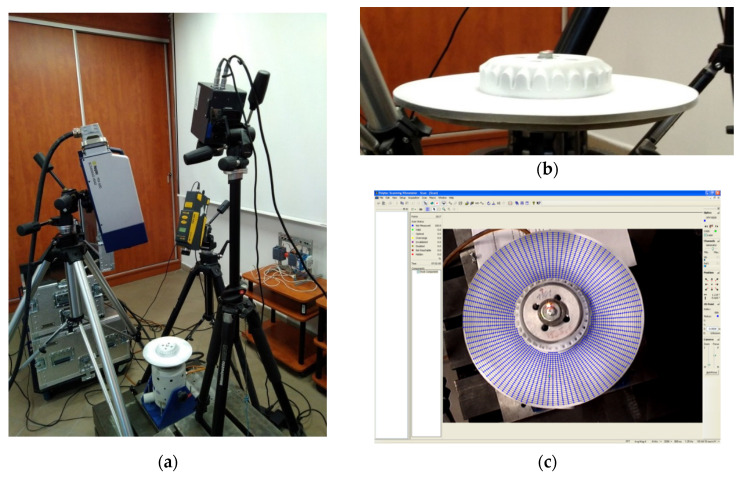
The experimental setup for vibration testing of PMC disks: (**a**) General view; (**b**) The view of the tested disk covered by the reflective powder; (**c**) The view from the vibrometer’s camera with the defined grid of measurement points.

The testing was performed using the test setup applicable for modal analysis of structures. The tested disks were mounted on the electrodynamic shaker TIRA^®^ TV-51120 (TIRA GmbH, Schalkau, Germany) connected to the amplifier TIRA^®^ B2 500 (TIRA GmbH, Schalkau, Germany) with a bolt screwed through their hubs. To ensure appropriate reflectivity, the tested surfaces were covered by the ARDROX^®^ 9D1B (Chemetall GmbH, Soissons, France) reflective powder (see Figure 4b).

The measurements were performed using two laser Doppler vibrometers (LDVs): the scanning LDV Polytec^®^ PSV-400 with the control unit Polytec^®^ PSV-W-400 (Polytec GmbH, Waldbronn, Germany) used for the measurements of the vibration velocity of the tested disks and the point LDV Polytec^®^ PDV-100 (Polytec GmbH, Waldbronn, Germany) focused on the fixing screw head and used in this study for the acquisition of the reference vibration signal. The complete experimental setup is presented in Figure 4a. The infrared camera visible in this figure was used during the tests with the SHVT technique described in detail in Section 2.2.3.

The modal analysis was performed by excitation of the tested disks with the pseudo-random noise signal in the frequency band of 1.25–3400 Hz and a resolution of 1.25 Hz for the larger disk, and 700–2200 Hz with a resolution of 1 Hz for the smaller disk. The frequency bands were defined based on the previously performed initial tests and the limitations in these bands were introduced to reduce the time of testing. The limitation of a frequency band for the smaller disk was applied due to the lack of excited eigenfrequencies with sufficiently high magnitude. The same rule was applied to define the upper limits for the frequency bands for both disks, i.e., with the higher frequencies, the determined eigenmodes were of low amplitude and proper identification of mode shapes was not possible due to biasing by noise. Four averages of the resulting frequency response functions (FRFs) were performed for each measurement point to reduce measurement errors. To identify structural flaws, dense grids of measurement points were defined (see Figure 4c for instance): 2617 and 2481 points for the larger and smaller disks, respectively, both in the polar coordinate system.

#### 2.2.2. Infrared Thermography

**Figure 5:**
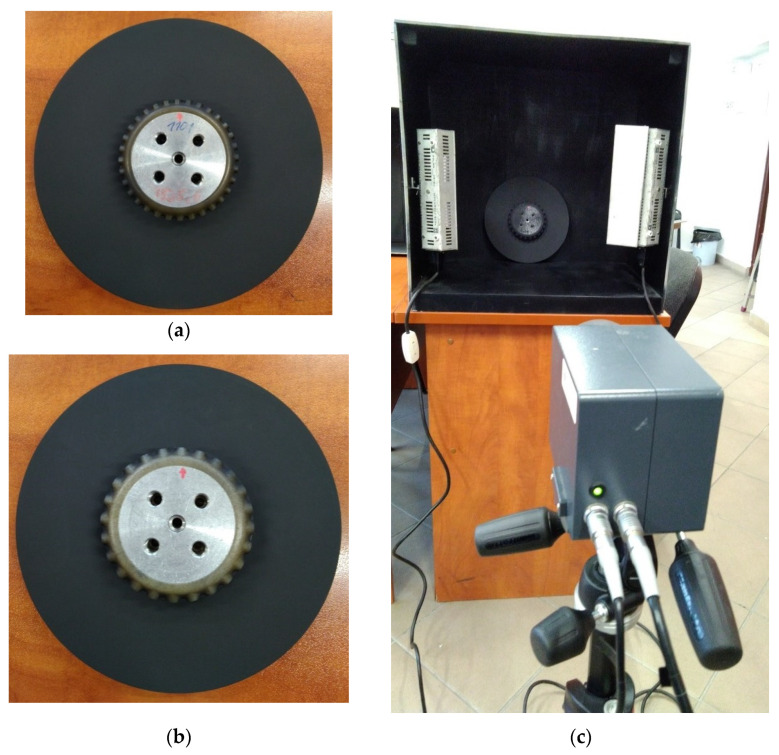
The experimental setup for IRT testing of PMC disks: (**a**,**b**) Larger and smaller disks covered with black enamel; (**c**) The general view of the experimental setup.

Prior to the IRT testing, the composite disks were covered with black matt heat-resisting enamel manufactured by Dragon Poland Sp. z o.o. (Cracow, Poland) to ensure an appropriate thermal emissivity of the tested surfaces. The testing was performed on the smooth surface of the disks. The disks after painting are presented in Figure 5a,b.

The disks were placed in a chamber covered with black matt paint to eliminate the influence of the surrounding heat sources and minimize reflections. The chamber was equipped with two lamp radiators with a power of 1 kW placed on both sides of the chamber and used for the thermal excitation of the disks (see Figure 5c). The preliminary testing was performed using the handheld infrared (IR) camera FLIR^®^ E6 (Flir Systems, Wilsonville, OR, USA). After the detection of damage, the main tests were performed using the InfraTec™ VarioCAM hr (InfraTec GmbH, Dresden, Germany) IR camera with increased thermal and spatial resolutions. The distance from the radiators to the tested disks was 0.4 m, while the distance from the IR camera to the disks was 1 m. The environmental temperature was 22 °C, while the emissivity of the disks was set to 0.9. The tests were performed using the transient IRT technique with a heating duration of 5 s. The surface temperature of the tested disks increased up to ca. 45 °C. After heating, the thermal response of the disks was registered using the InfraRec™ IRBIS 3 software. The thermograms were collected for each tested disk during the cooling stage (after removing the thermal excitation) with a framerate of 5 frames per second.

#### 2.2.3. Self-Beating Based Vibrothermography

The SHVT testing was performed on the disks previously covered by the black matt heat-resisting enamel (see Section 2.2.2) and after drying up also with the reflective powder (see Section 2.2.1). The SHVT testing consisted of two steps. First, the modal analysis was performed using the same test setup as presented in Figure 5a and with the same parameters of testing as described in Section 2.2.1. The selected natural frequencies with the highest magnitudes of vibration identified after performing the modal analysis were then used for the mechanical excitation of the disks. The harmonic signals were used to excite mechanical vibrations at the determined natural frequencies of the disks with a force of ±200 N. Taking into account several mode shapes is essential, since it allows for the consideration of the whole surface of a tested disk since the maximal temperature increase is expected in the location of maximal magnitudes of vibration and near the fixture. The increasing self-heating temperature is directly related to mechanical stress during vibration excitation. The thermal response of the tested disks was observed at this type of excitation for 2 minutes in each case until the observed stabilization of surface temperature. The framerate of acquisition of the thermograms was 5 frames per second. More information on the details of the method can be found in [49,50].

#### 2.2.4. Ultrasonic Testing

**Figure 6:**
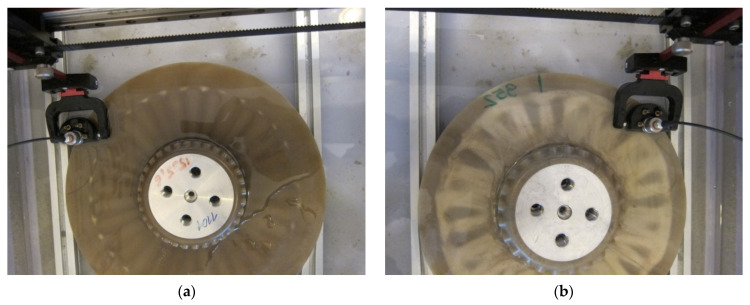
The testing procedure of (**a**) larger and (**b**) smaller disks using UT technique.

The UT of the composite disks was performed using the Boeing MAUS^®^ V automated scanning system (Chicago, IL, USA) with the 5 MHz (6 mm) testing probe, which provides the best results for the composite structures as a result of a compromise between the ability to reach high resolution and damping of the tested structures. During the test, the disks were immersed in water to obtain a good coupling between the probe and the tested disks as well as controlled attenuation of the sound path in water. The testing process using the UT technique is presented in Figure 6.

The initial verification was performed in the A-scan mode. After successful verification, the testing was performed in the C-scan mode with a spatial resolution of 0.1 mm. Due to the geometry of the objects, the research was carried out only within the flat part of the disks without data registration in the axial part. The tests were carried out in four mutually overlapping areas due to the raster scanning mode. Both Time-of-Flight (ToF) and amplitude modes were initially taken into consideration during scanning, however, after the analysis of the preliminary results, the amplitude mode was assumed for further studies due to its better performance in the identification of the internal lower density areas.

#### 2.2.5. X-ray Computed Tomography

The XCT testing was performed using the phoenix v|tome|x m 300 industrial CT scanner (GE^®^ Measurement & Control Solution, Billerica, MA, USA) with a maximum power of 500 W at 300 kV voltage and the GE^®^ detector of DXR type with a 400 × 400 mm active area. The spatial resolution of the resulting tomograms was 105 μm^3^, which was enough to observe and quantify the lower density areas in the tested disks. The obtained tomograms were processed using the VGStudio MAX software (Volume Graphics GmbH, Heidelberg, Germany) to achieve a desired contrast and sharpness as well as to quantify the lower density areas in the tested disks and to compare the quantified manufacturing defects with the results of other NDT techniques considered in this study.

## 3. Results

### 3.1. Damage Identification Based on Modal Analysis

**Figure 7:**
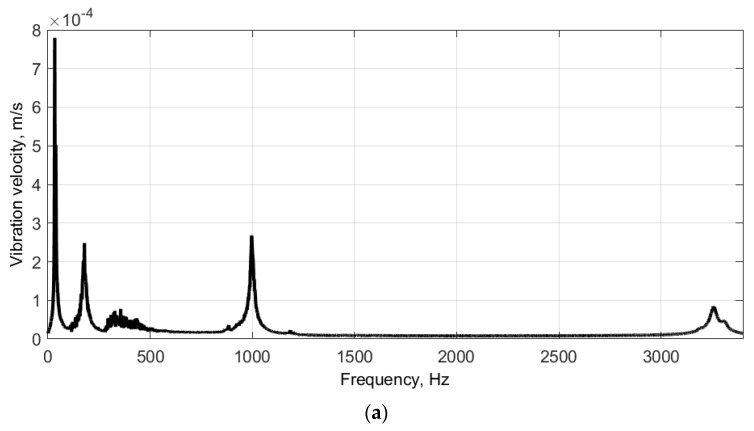

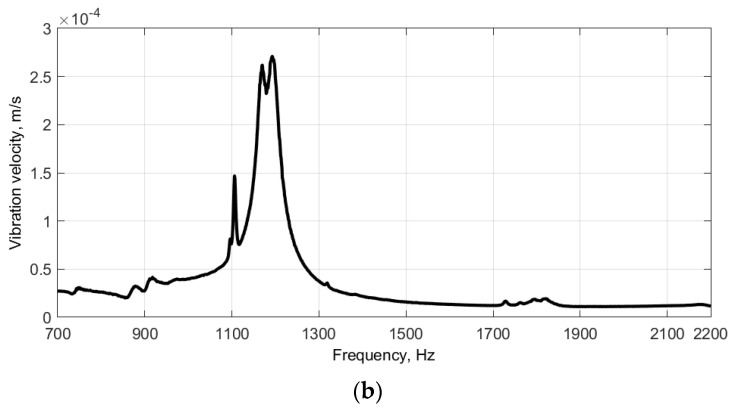
The FRFs of the larger (**a**) and smaller (**b**) disks.

**Figure 8:**
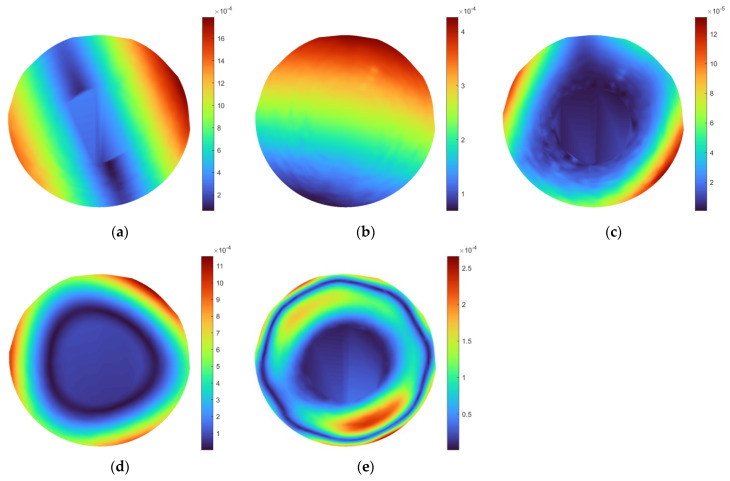
The interpolated mode shapes of the larger disk: (**a**–**e**) subsequent considered mode shapes.

**Figure 9:**
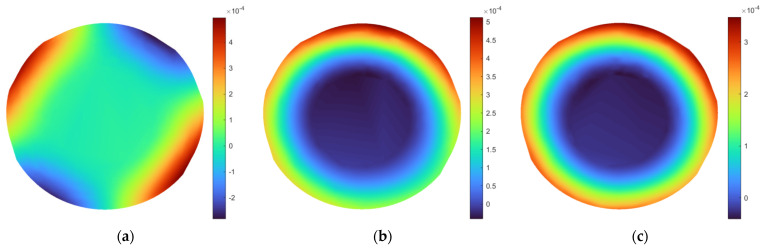
The interpolated mode shapes of the smaller disk: (**a**–**c**) subsequent considered mode shapes.

As a result of modal testing of the disks, the FRFs were obtained, which are presented in Figure 7. The values of the natural frequencies determined from the FRFs are presented in Table 1. The corresponding mode shapes of the tested disks are presented in Figure 8 and Figure 9, respectively. Due to the irregular distribution of the measurement points on the boundaries of the defined grids (see Figure 4), the interpolation was applied to the resulting mode shapes for the visualization purpose. According to this, the central part, i.e., the part without the defined grid of measurement points (see Figure 4), was not taken into consideration in the analysis of results.

Analyzing the obtained mode shapes, which correspond to the identified natural frequencies of vibration, it can be noticed that, in general, the highest magnitudes of vibration, represented by colors in Figure 8 and Figure 9, are observable on the exterior boundaries of the disks. This is connected with the presence of stiffening ribs on the backside of the disks and increasing the height of these ribs closer to the hubs. This, as a consequence, caused a gradual increase in the stiffness of the disks towards the hub. Nevertheless, some disturbances in mode shapes are observable for the larger disk for modes 2 and 3 (see Figure 8b,c), which may point to the presence of lower density areas in this location.

**Figure 10:**
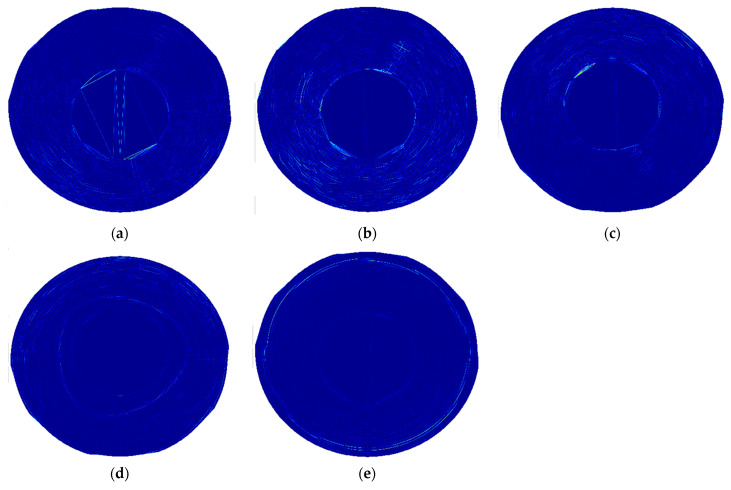
The post-processed mode shapes of the larger disk: (**a**–**e**) subsequent considered mode shapes.

**Figure 11:**
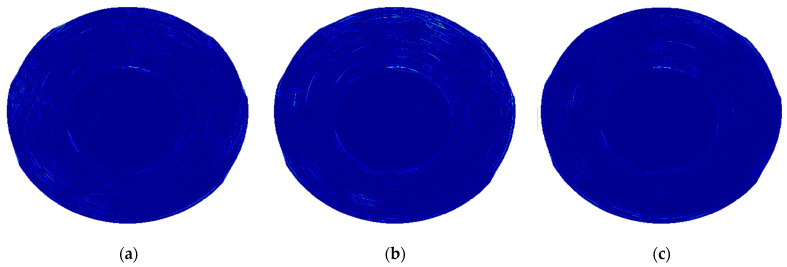
The post-processed mode shapes of the smaller disk: (**a**–**c**) subsequent considered mode shapes.

To enhance the visibility of the identified disturbances in mode shapes, their post-processing was performed using the non-decimated discrete wavelet transform, which avoids the reduction of the size of the initial grid by omitting the decimation operation. The single-level decomposition was performed using the second-order B-spline wavelet, which was selected according to the previous analysis described in [61]. During the analysis, three types of detail coefficients (horizontal, vertical, and diagonal) were considered due to the expectation of these coefficients consisting of useful diagnostic information. Then, the absolute values of the detail coefficients were added up to represent the results for each considered mode in a single plot. The processed mode shapes of the disks are presented in Figure 10 and Figure 11, respectively. The colors of these figures represent the magnitudes of wavelet coefficients, representing in turn, the detected flaws. These locations are represented by lighter colors, other than blue.

As it can be observed from the above figures, the high-magnitude coefficients are observable in the possible locations of lower density areas, however, they do not allow stating about unequivocal damage localization and identification.

### 3.2. Infrared Thermography

**Figure 12:**
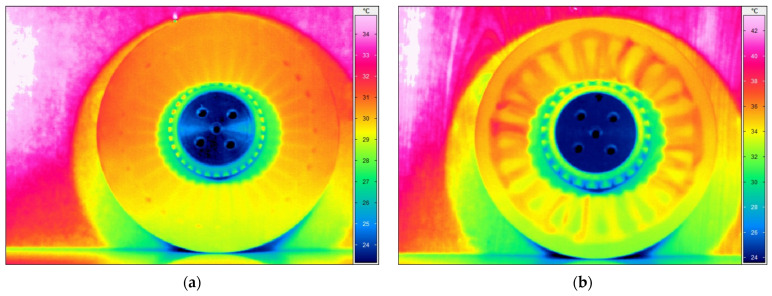
The representative thermograms of the (**a**) larger and (**b**) smaller disks.

The tests performed according to the methodology described in Section 2.2.2 allowed for collecting a series of thermograms for both tested disks. The representative thermograms are presented in Figure 12, where the higher temperature was registered in the locations of the lower density areas detected in the tested disks.

In both cases, the lower density areas are clearly visible. Due to the different depths of the identified lower density areas, which is a result of the manufacturing process, the representative thermograms originate from various periods of cooling. For the larger disk (Figure 12a), the strengthening ribs are recognizable and numerous locations of lower density areas are visible as hot spots on the perimeter of the ribbing flattening. The identified lower density areas are in agreement with the pattern of defects visible in Figure 3a. Similarly, in the case of the smaller disk, extensive lower density areas are observed near the surface of the non-ribbed side, which is in agreement with the whitening areas observable in Figure 3b. The IRT testing confirmed the capability of identification of internal lower density areas in the tested disks.

### 3.3. Self-Heating Based Vibrothermography

**Figure 13:**
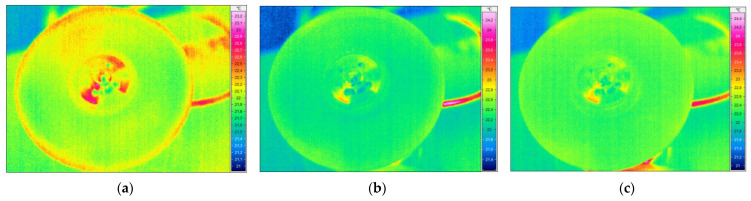
The selected thermograms obtained during the SHVT testing of the larger disk with excitation frequencies of (**a**) 33.75 Hz; (**b**) 178.75 Hz; (**c**) 997.5 Hz.

**Figure 14:**
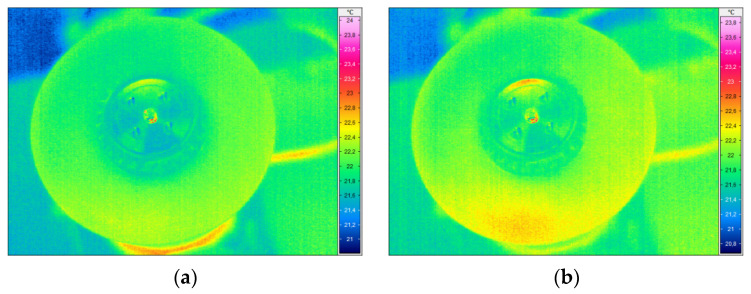
The selected thermograms obtained during the SHVT testing of the smaller disk with excitation frequencies of (**a**) 1106 Hz; (**b**) 1170 Hz.

The excitation of the larger disk was performed with three selected natural frequencies with the highest magnitudes of vibration: 33.75 Hz, 178.75 Hz, and 997.5 Hz (see FRF presented in Figure 7a and the corresponding mode shapes presented in Figure 7a,b,d). The difference in the last considered natural frequency (cf. Table 1) was probably a result of the differences in momentum during tightening the fixing screw. For the excitation of the smaller disk, two natural frequencies were used: 1106 Hz and 1170 Hz, which are in agreement with the measurements during the modal analysis (see Table 1). The FRF and the corresponding mode shapes are presented in Figure 7b and Figure 9a,b, respectively. The selected thermograms for the particular natural frequencies are presented in Figure 13 and Figure 14.

The slight increase in temperature due to the self-heating effect was observed in both cases, however, due to the high stiffness in the ribbed region of both disks, the resulting stress due to the mechanical excitation was too small and the detection of lower density areas was not possible using this approach. 

### 3.4. Ultrasonic Testing

**Figure 15:**
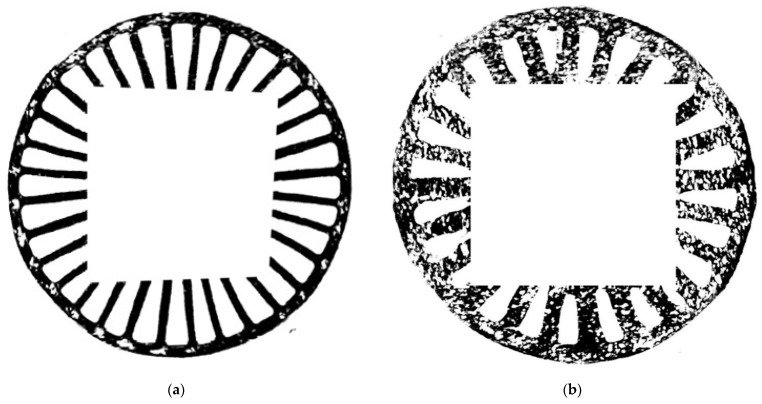
The amplitude mode merged UT C-scans of (**a**) larger and (**b**) smaller disks.

Figure 15 shows the final results of UT in amplitude mode after merging four raster scans.

In Figure 15a, the dark areas correspond to the ribbing of the larger disk, representing the areas in which the signal is scattered, mainly due to the geometry of the tested element. The background signal from the rest of the disk was uniform and not indicative of any pores, inclusions, or lower density areas. Areas identified and labeled as the lower density ones are mainly visible on the rim of the disk’s edge as areas of a contrasting white tinge. These areas, due to the gradient change in the stiffness of the element, cause signal reflections of varying intensity, resulting in areas of variable contrast, visually consistent with the whitening observable in Figure 3a. The internal ribbing shows a fairly uniform reflected amplitude distribution, which also indicates no defects in this area.

As it can be seen from Figure 3b, the smaller disk contains areas where the lower density areas are visible not only on the rim of the disk but also on the area under ribbing and in the areas of the disk between the ribs. In this case, the larger signal scattering is observed. A comparison of the UT results for larger and smaller disks reveals significantly wider signal attenuation in the area of the ribs and rim of the smaller disk. The signal attenuation is due to the occurrence of lower density areas in the rib areas. These areas cause both signal absorption due to the rib geometry and the residual reflection from the lower density areas. Depending on the location, the reflected amplitude distribution of the lower density areas varies even within 6 dB, which may indicate a different intensity of the occurrence of this damage.

### 3.5. X-ray Computed Tomography

**Figure 16:**
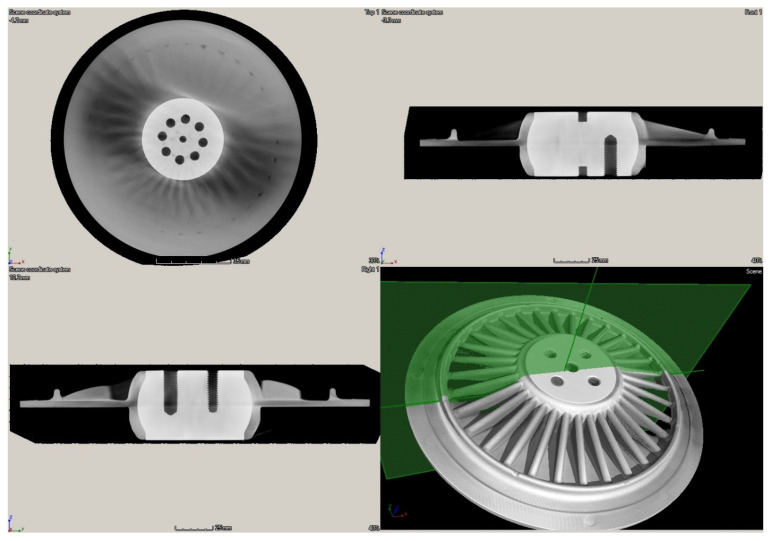
The general 3D view with cross-sections of the tomogram of the larger disk.

**Figure 17:**
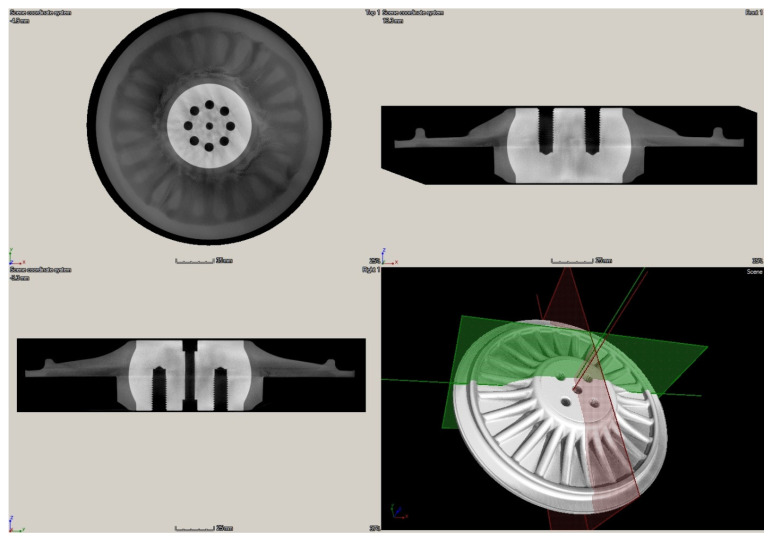
The general 3D view with cross-sections of the tomogram of the smaller disk.

The obtained scanning results using the XCT technique are presented in Figure 16 and Figure 17 for both tested disks. Thanks to the possibility of 3D mapping of an internal structure of the disks, it was possible to identify and quantify internal lower density areas and validate the previously presented NDT results within this section. As it can be observed in Figure 16, the larger disk consists of lower density areas on the rim of the disk’s edge, which was also detected with IRT (see Figure 12a) and UT (see Figure 15a) techniques. No other lower density areas were detected in this disk.

In the case of the smaller disk, the numerous lower density areas were detected in the flat subsurface region of the disk, which coincides with the previously obtained results with IRT (see Figure 12b) and UT (see Figure 15b) techniques. This validation proved the effectiveness of IRT and UT techniques in the detection of lower density areas in the tested disks, however, to evaluate the performance of the mentioned NDT techniques in damage identification, in particular for further application in PI and QC, an analysis of the depth of the detected lower density areas from tomograms was necessary.

**Figure 18:**
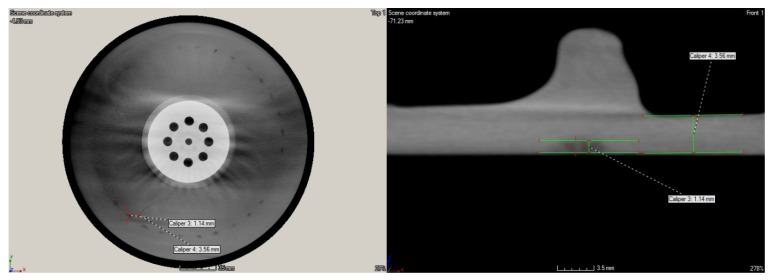
The selected detailed views of the tomogram of the larger disk with dimensioned lower density areas.

**Figure 19:**
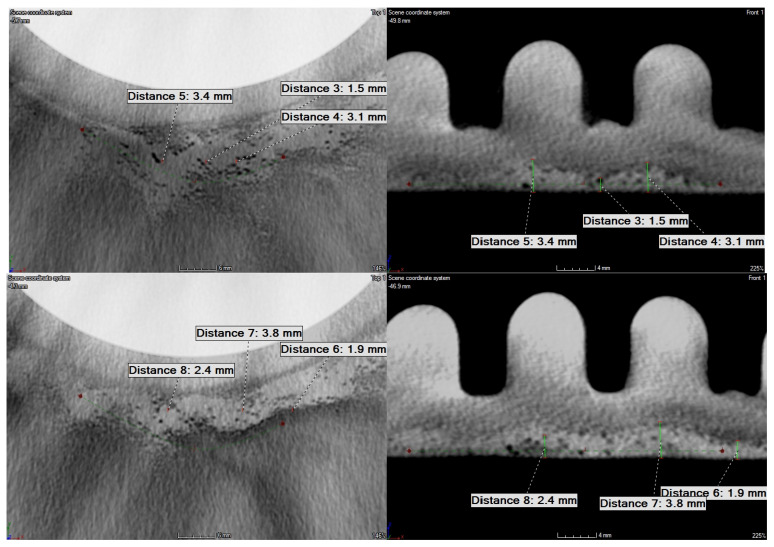
The selected detailed views of the tomogram of the smaller disk with dimensioned lower density areas.

The detailed analysis of the obtained tomograms allowed for quantifying the depths of the lower density areas in both tested disks. The analysis confirmed that none of such areas are present in the ribbing regions, while all detected lower density areas are located in the flat plate-like part of disks. The detected lower density areas on the rim of the disk’s edge in the larger disk were on the level of ca. 1 mm (see Figure 18). In the case of the smaller disk, the extensive lower density areas were of various depths, which did not exceed 4 mm. The exemplary images with dimensions are presented in Figure 19.

## 4. Discussion and Recommendations for NDT Inspection in Industrial Conditions

### 4.1. Discussion on the Testing Results

Damage identification in thick composite structures is a challenging task for a few reasons. It consists of the possible location of several failure modes at different depths and orientations; quality of manufacturing, which influences NDT techniques performance (especially on ultrasonic); material properties (layers orientation, thermal, and mechanical properties of a polymer used for a composite’s matrix); the geometry of a tested structure or element, which may limit some capabilities of some techniques (such as ultrasonics); NDT operator qualification, and others.

Taking these reasons into account enforces a careful selection of the inspection technique for a particular inspection task. In this paper, an application of several NDT techniques was presented to highlight its applicability for the identification of lower density areas and porosity.

The performed research studies using the method of damage identification based on a modal analysis allowed for the determination of FRF and the corresponding mode shapes of the tested disks. The testing technique is based on conducting a modal analysis of the tested disks with a densified grid of measurement points, and then, based on the identified mode shapes, identification of anomalies with the use of post-processing methods based on wavelet analysis. An important factor during the implementation of this technique is the appropriate preparation of the surface by ensuring the level of the signal representing the appropriate reflection of a laser beam from the surface, which directly affects the quality of the acquired signal. The measuring ranges for the main and the reference signals should be set in such a way that they do not cause overestimation. The laser beam should also be properly focused, which affects the quality of the acquired signal, both the main and the reference one. At the post-processing stage, the type of transform and the basis function should be selected appropriately, so that the sensitivity of the algorithm to anomalies in mode shapes is as high as possible. The crucial factor affecting the sensitivity of vibration-based approaches to internal defects is the stiffness of a tested structure, which directly corresponds with its thickness. As it was observed in one of the previous studies [62], where a similar vibration-based approach was applied to damage identification in aircraft structures, identification of flaws in thick and locally stiff structures could be problematic, which is confirmed in the present study. Based on the results of the performed research studies and the previous experience in using this approach, one can state the following:

- the method based on modal analysis using the laser vibrometry measurement technique with post-processing of mode shapes works well for structures that are flexible enough and/or with sufficiently large defects, which would allow potential defects to affect changes in the mode shapes; these changes do not have to be directly identifiable in their mode shapes, the technique allows its application to even minor defects;
- the method based on modal analysis using the laser vibrometry measurement technique is a non-contact method and therefore it is effective for testing elements with complex geometry;
- based on the presented results, it can be seen that too high of stiffness in the location of defects prevents their successful and unequivocal detection;
- in the light of the research carried out within this study, it can be concluded that the approach based on modal analysis with further post-processing of mode shapes may find potential application in the identification of defects such as surface defects, delamination, foreign objects inclusions, and others, in particular for elements of relatively low stiffness, including thin-walled elements.

As in the method based on modal analysis (which is the first stage of the SHVT approach), the decisive factor in such tests is the stiffness of the tested object. During the SHVT tests, in addition to the above-mentioned factors regarding the modal analysis, the high-frequency resolution of the obtained FRF is also important due to the need to stimulate the tested element at a frequency as close as possible to a given resonance frequency, since it is important to stimulate the self-heating effect. This amplitude of vibration is directly related to the stresses that are, in turn, related to the amount of heat generated in the tested structure. According to the results of wide research studies on the criticality of the self-heating effect [64], the magnitude of vibrations should not be high, which might cause irreversible processes in the tested structure, which may lead to damage initiation. The observations from the obtained results can be summarized as follows:

- as a result of the conducted tests, it was not possible to detect damage in the form of lower density areas in the tested disks, however, in one of the elements it was possible to stimulate the self-heating effect at the edge, which allows for further consideration of the method in the study of other polymer and composite structures;
- the SHVT technique is a non-contact method and is therefore effective for testing elements with complex geometry;
- the SHVT technique is suitable for the detection and identification of surface defects, delamination, foreign object inclusions, etc., for elements with relatively low stiffness, including thin-walled structures. In particular, defects and damage located in the locations of stress concentration will be well detectable in this case due to the aforementioned relationship of stress and the amount of heat generated in the tested structure;
- the technique, apart from the limitations resulting from stiffness (which was observed in the conducted tests), has a limitation regarding the tested materials: SHVT allows testing only objects made of polymeric materials or containing polymers, such as polymer matrix composites, due to the fact that that the self-heating effect occurs due to the viscoelastic properties of the material from which the test object is made and subjecting it to forced cyclic loads.

The use of both modal analysis and SHVT techniques gives a large spectrum of structural flaws visualization and potential stiffness changes, which may be very efficient for the identification of numerous types of defects and damage. The structure of lower density areas in the presented results does not sufficiently respond to both methods of excitation, therefore using these methods for detection and identification of lower density areas is not the most efficient approach mainly due to the low global changes in the stiffness.

The transient thermography technique allows for the detection and identification of defects and damage in various types of materials, including polymeric, composite, metal materials, and their various combinations. An important factor in IRT tests is the appropriate preparation of the tested surfaces (i.e., removing all possible reflections) and limiting the exposure of elements against external heat sources and possible reflections. Depending on the tested materials, the parameters of IRT testing may differ significantly, e.g., time of exposure to thermal forcing of the tested surfaces. As previously stated, the crucial factor influencing the detectability of flaws is the thickness of a tested structure. However, the sensitivity of IRT is suitable for the detection and identification of subsurface defects, such as lower density areas, which was confirmed in numerous previous studies (see e.g. [65,66,67]), including inspection of thick composite structures, such as wind turbine blades. The following observations were made during testing using this technique:

- the method of transient thermography is well suited to the detection of surface and subsurface defects and damage, which was confirmed in the present studies, however, its sensitivity significantly decreases with an increase in distance to flaws from the inspected surface, and the closer flaws may mask the underneath ones (see Figure 12);
- an advantage of the IRT technique is the possibility of performing contactless measurements, which makes it possible to test elements with complex geometry;
- this method is characterized by a high speed of testing, which enables a rapid and less costly inspection of elements, e.g., as part of manufacturing quality control;
- the effectiveness of detecting a defect or damage largely depends on the thickness of the tested element and the thermal properties of the material of the tested element—in the case of thick structures, the quality of the obtained results and the detectability may be significantly limited. From the collected results it is visible that identification of lower density areas is possible, however, limited to locations close to the inspected surface. Deeper located flaws may not be visible due to the thermal energy dissipation and small temperature gradients to conclude the presence of a flaw.

The most promising results are obtained with the use of the UT method. This method allows evaluating the presence of lower density areas based on the possibility of ToF as well as amplitude data analysis. A combination of these two approaches gives the possibility for evaluation flaw location and geometry; size estimation based on selected criteria (e.g., 6 dB signal drop); damage type quantification (based on signal criteria); possibility of damage comparison (using additional image processing). The presented results allowed visualizing not only the subsurface defects, such as IRT but also defects located in the deeper parts of tested disks. This is due to a fact that the penetration ability of an ultrasound beam is better than in the case of a heatwave for the investigated structures. However, it is important to mention that UT reveals less sensitivity to internal defects and damage located close to the surface, which was broadly discussed in [68]. This implies a necessity of a combination of the analyzed NDT techniques to improve the effectiveness and validity of PI and QC of polymeric and PMC elements and structures.

Due to the complex nature of the structural composition of composite materials and the desired sensitivity of a technique, an important factor is not only the selection of the appropriate measurement technique but also the proper selection of test parameters (measurement frequency, type, and size of the sensor). The type of structure of the tested material during UT is essential. The use of different fiber structures (reinforcement) of composite materials or polymers (e.g., resins) can significantly affect the attenuation of the acoustic wave in such a material (through the phenomenon of diffusion and reflection). Another important element is the preparation of the surface and its geometry. High porosity surfaces may make testing difficult due to the lack of proper acoustic coupling. On the other hand, elements with complex geometry, due to the scattering phenomenon, can significantly hinder the signal analysis process or make it impossible to test, e.g., using the reflection technique. Nevertheless, except for the XCT, only UT is confirmed to be suitable for testing thick composite structures according to the numerous authors (see e.g., [6,69]). This also found a confirmation in the obtained results in the current study, where the location and distribution of the lower density areas observed in C-scans (see Figure 15b) significantly differs from the whitening areas observable in Figure 3b, which allows for presuming that the lower density areas identified with UT consist of not only near-surface internal defects but also those located deeper in the tested structures. The analysis of the UT results allowed drawing the following observations:

- automated UT allowed for successfully identifying lower density areas in the tested disks. Within this study, the resin poor areas were detected and confirmed, which confirms the appropriateness of this technique in the detection and identification of lower density areas;
- automated UT tests allow for the visualization of the geometry of the tested element–determining the correctness of its implementation and determining the size and location of flaws. Moreover, when using signal normalization, it is possible to use time imaging, e.g., to determine the depth of damage;
- the UT testing has several limitations such as the necessity of ultrasonic signal coupling, difficulties in an inspection of thick materials due to high acoustic attenuation, limitations connected with the geometry of the inspected element, a strong influence of the entry surface condition which may influence on signal propagation, etc.

The performed validation studies using XCT revealed excellent mapping of the lower density areas in the tested disks. The obtained results confirmed the sensitivity of both UT and IRT techniques to subsurface lower density areas and the ability of their proper identification for PI and QC needs. Although XCT provides a tool for a comprehensive mapping of lower density areas, performing PI and QC in an automated way is difficult using this technique. The scanning procedure with desirable resolution takes usually from tens of minutes to hours, it cannot be automatized due to the specificity of the testing facility and conditions as well as the costs of scanning are relatively higher compared to testing with other techniques considered in this study. Moreover, the lower density areas are a very special type of defect, which makes it difficult to visualize them using the differences in contrast as it is performed with porosity. In the case of lower density areas, the lower density areas cannot be labeled as it is performed in the case of porosity, where the entrapped air bubbles create a sharp difference with the material. This deficiency is an additional argument on the non-coincidence of the XCT technique with the requirements of PI and QC in industrial conditions.

### 4.2. Recommendations for Industrial Applications of NDT Techniques

As one can see, the approach for the inspection of the tested structures from the industrial point of view is a challenging task. An approach for inspection should be carefully selected taking into account numerous factors, such as expected failure modes, thickness, and geometry implications as well as several constraints including PI and QC demands as well as economic factors.

The following study discusses the applicability of the selected NDT techniques for the identification of lower density areas in composite disks, which makes this study case-specific, and according to the summaries of observations presented in Section 4.1 for the pre-selected NDT techniques, it can be stated that the planning of NDT is strictly case-specific. As it can be concluded from the obtained results, the IRT and UT techniques seem to be the most appropriate for the identification of internal lower density areas in the tested composite disks due to their sensitivity to flaws and relatively low inspection times. Moreover, the IRT and UT techniques are complementary to each other considering the character of the investigated type of defects. The analyzed defects are related primarily to the manufacturing process, where these techniques can be used for an automated QC ensuring meeting the appropriate operational requirements and safety of the considered disks.

The automation QC in many cases is a key direction of the development of such industrial systems, especially in increasing production volumes. The IRT and UT techniques are good candidates for inspection automation, primarily due to the possibility of fast testing with little setup and initial preparation of tested elements. Moreover, both techniques are portable and easily adjustable to various environmental conditions. The capability of automation of UT testing is presented in this paper by performing research using the automated UT system. The automated systems based on this technique are described in numerous publications (see e.g., [70,71,72]). Similarly, the automation of IRT inspections is also reported (see e.g., [73,74,75]). It is therefore recommended to apply a combination of IRT and UT techniques since IRT is sensitive to defects and damage located close to a surface, while UT reveals a great sensitivity to defects and damage located deeper. Considering the physics of lower density areas in polymeric and PMC structures and their related dimensions, the proposed approach can identify such types of defects with a sensitivity comparable to XCT. The performed XCT tests verified the performance of both techniques, i.e., based on the obtained results it is possible to state that the mentioned combination of NDT techniques may successfully substitute XCT testing, being faster and more economically efficient. Moreover, the proposed approach can overcome the second major limitation of XCT, namely, limits on the dimension of a tested object.

To improve the sensitivity of the considered NDT techniques, post-processing is widely used, both in UT and IRT-based inspections, Although requiring more advanced systems, the post-processing can be also incorporated into an automated NDT system. Moreover, the presented results indicated the possibility of the multimode approach for the determination of voids’ presence in structures. The use of such approaches opens the possibility for data fusion, which may allow for the enhancement of damage identification. Finally, the promising and currently investigated direction of development of PI and QC approaches based on NDT techniques is their combination with methods of artificial intelligence, which makes it possible to achieve even higher sensitivity to various types of defects and damage (see e.g., [76,77]). However, in industrial applications there are demands connected with method qualification, relevant standards existence as well as method reliability and repeatability.

## 5. Conclusions

The intention of this paper was to present the performance of the selected NDT techniques based on the case study of the identification of internal lower density areas in composite disks, discuss the applicability of the selected NDT techniques in industrial conditions, and propose an effective tool based on a combination of various NDT techniques for fast and effective PI and QC of investigated elements with a possibility of automation of an inspection process. The NDT experiments confirmed the feasibility of the selected methods in the identification of internal lower density areas in the tested composite disks, which was additionally verified using XCT results as a reference, while the wide discussion on the properties, advantages, and deficiencies of the considered NDT techniques allowed selection of the most appropriate ones for the investigated case. The performed experimental studies and further analysis of the performance of particular NDT techniques indicated that two of the considered NDT techniques, namely, UT and IRT, can successfully detect the lower density areas in the tested composite disks and meet the industrial requirements in sensitivity and accuracy of identification of lower density areas, portability and universality of the testing environment, easiness of implementation of inspections, and a possibility of inspection automation. The improvement of the inspection performance can be achieved by a proposed combination of these techniques, which provides a reliable inspection methodology being an alternative to the XCT testing, which is time-consuming and costly. The obtained experimental results allowed for confirming the sensitivity of the selected methods, comparing the obtained results to those available in literature as well as to propose recommendations on selection and application of these NDT techniques in detection of lower density areas in the light of its application in industrial conditions for quality control.

## Figures and Tables

**Table 1:** Natural frequencies of vibration of the tested disks corresponding to mode shapes presented in Figure 8 and Figure 9.

Larger Disk	Smaller Disk
33.75 Hz	1106 Hz
178.75 Hz	1170 Hz
881.25 Hz	1193 Hz
996.25 Hz	
3261.25 Hz	

## Data Availability

The data that support the findings of this study are available upon request from the authors.
